# Replication of avian, human and swine influenza viruses in porcine respiratory explants and association with sialic acid distribution

**DOI:** 10.1186/1743-422X-7-38

**Published:** 2010-02-16

**Authors:** Sjouke GM Van Poucke, John M Nicholls, Hans J Nauwynck, Kristien Van Reeth

**Affiliations:** 1 Laboratory of Virology, Faculty of Veterinary Medicine, Ghent University, Salisburylaan 133, 9820 Merelbeke, Belgium; 2 University of Hong Kong, Department of Pathology, Room 314, Pok Fu Lam Road, Hong Kong SAR, China

## Abstract

**Background:**

Throughout the history of human influenza pandemics, pigs have been considered the most likely "mixing vessel" for reassortment between human and avian influenza viruses (AIVs). However, the replication efficiencies of influenza viruses from various hosts, as well as the expression of sialic acid (Sia) receptor variants in the entire porcine respiratory tract have never been studied in detail. Therefore, we established porcine nasal, tracheal, bronchial and lung explants, which cover the entire porcine respiratory tract with maximal similarity to the *in vivo *situation. Subsequently, we assessed virus yields of three porcine, two human and six AIVs in these explants. Since our results on virus replication were in disagreement with the previously reported presence of putative avian virus receptors in the trachea, we additionally studied the distribution of sialic acid receptors by means of lectin histochemistry. Human (Siaα2-6Gal) and avian virus receptors (Siaα2-3Gal) were identified with *Sambucus Nigra *and *Maackia amurensis *lectins respectively.

**Results:**

Compared to swine and human influenza viruses, replication of the AIVs was limited in all cultures but most strikingly in nasal and tracheal explants. Results of virus titrations were confirmed by quantification of infected cells using immunohistochemistry. By lectin histochemistry we found moderate to abundant expression of the human-like virus receptors in all explant systems but minimal binding of the lectins that identify avian-like receptors, especially in the nasal, tracheal and bronchial epithelium.

**Conclusions:**

The species barrier that restricts the transmission of influenza viruses from one host to another remains preserved in our porcine respiratory explants. Therefore this system offers a valuable alternative to study virus and/or host properties required for adaptation or reassortment of influenza viruses. Our results indicate that, based on the expression of Sia receptors alone, the pig is unlikely to be a more appropriate mixing vessel for influenza viruses than humans. We conclude that too little is known on the exact mechanism and on predisposing factors for reassortment to assess the true role of the pig in the emergence of novel influenza viruses.

## Background

Pigs are important natural hosts for influenza A viruses, which are a major cause of acute respiratory disease. Influenza viruses of H1N1, H3N2 and H1N2 subtypes are enzootic in swine populations worldwide. Most of these swine influenza viruses are the product of genetic reassortment between viruses of human and/or avian and/or swine origin and their phylogeny and evolution are complex [1-3]. The swine influenza viruses circulating in Europe have a different origin and antigenic constellation than their counterparts in North America or Asia and within one region multiple lineages of a given subtype can be present [4,5]. Although natural infections of pigs with avian [6-10] or human influenza viruses [11,12] also occur, these viruses were rarely capable of establishing themselves as a stable lineage in pigs without undergoing genetic adaptation [13].

Because sialic acids (Sia) with α2,6 and α2,3 linkages to galactose (receptors preferred by human and avian influenza viruses respectively) were identified in the porcine trachea, pigs have been implicated as intermediate hosts or as mixing vessels for reassortment [14-16]. As such, co-infection with human and AIVs or with human, swine and AIVs could lead to the emergence of new influenza viruses with a pandemic potential. On the other hand, the generation of pandemic influenza viruses in pigs appears to be a rare and complex process, and the 2009 H1N1 influenza virus is the first pandemic virus that is almost certainly of swine origin.

Though experimental *in vivo *studies [17-21] have confirmed the susceptibility of pigs to both avian and human influenza viruses, they also point towards a strong species barrier as virus titers obtained from the respiratory tract and from nasal swabs were invariably lower for the heterologous viruses than for typical swine influenza viruses. In addition, all AIVs examined failed to transmit between pigs [22,23]. Limited *in vitro *studies, using either porcine tracheal organ cultures [24] or primary swine respiratory epithelial cell cultures (SRECs) [25] confirmed the lower susceptibility of the pig tissues to most heterologous viruses. In the SRECs, Busch and co-workers identified molecular differences in the HA gene which correlated with the divergence in infectivity.

However, the replication efficiencies of influenza viruses from various hosts as well as the expression of Sia receptor variants have never been compared at all levels of the porcine respiratory tract. For this purpose, we (1) established porcine nasal, tracheal, bronchial and lung explants covering the entire porcine respiratory tract with maximal similarity to the *in vivo *situation, (2) investigated the replication ability of avian, human and swine influenza viruses in all relevant parts of the respiratory tract and (3) analyzed the receptor distribution by means of lectin histochemistry.

## Results

### 1. Viability

The cilia on the epithelial cells of the nasal explants (NE) and tracheal explants (TE) continued beating for at least 72 h after sampling.

The percentages of ethidium monoazide bromide (EMA) and Terminal deoxynucleotidyl transferase mediated dUTP Nick End Labelling (TUNEL) positive cells in the four explant systems between 0 and 96 hours post culture (hpc) are shown in Table 1. Every result was the mean of 12 counts. The percentage of necrotic and apoptotic cells generally remained below 5% for NE and TE and below 10% for bronchial explants (BE) and lung explants (LE) during the entire period. There were only two exceptions: the TE at 24 hpc and the LE at 96 hpc.

**Table 1:** Viability of explant systems

	% EMA-positive cells at.....h of cultivation					% TUNEL-positive cells at.....h of cultivation				
	**0**	**24**	**48**	**72**	**96**	**0**	**24**	**48**	**72**	**96**

NE	0.3 ± 0.6	0.9 ± 1.4	0.2 ± 0.6	0.7 ± 0.6	0.5 ± 0.9	0.8 ± 1.0	0.5 ± 0.7	0.5 ± 0.7	0.8 ± 0.8	0.8 ± 1.0
TE	1.9 ± 1.4	0.6 ± 1.0	0.8 ± 1.5	0.8 ± 0.4	0.6 ± 1.1	1.1 ± 1.3	5.0 ± 2.1	0.9 ± 1.2	1.0 ± 1.4	1.1 ± 1.1
BE	1.7 ± 1.3	5.2 ± 1.8	1.6 ± 1.6	5.0 ± 2.1	3.0 ± 1.9	5.3 ± 1.6	5.0 ± 1.0	5.0 ± 2.1	3.0 ± 1.7	5.1 ± 1.1
LE	5.1 ± 2.8	4.4 ± 1.3	5.1 ± 2.4	5.1 ± 2.5	7.7 ± 1.4	3.7 ± 1.4	5.1 ± 1.2	5.0 ± 0.7	5.3 ± 1.6	10.0 ± 1.4

Mean percentages of apoptotic (TUNEL stained) and necrotic (EMA stained) cells in the four explant systems until 96 hours post cultivation.

Overall, it was concluded that the fluctuations of virus yields over time were a true reflection of virus replication since the proportion of dead cells in the explants showed little variation until at least 72 hpc.

### 2. Virus yield

**Figure 1:**
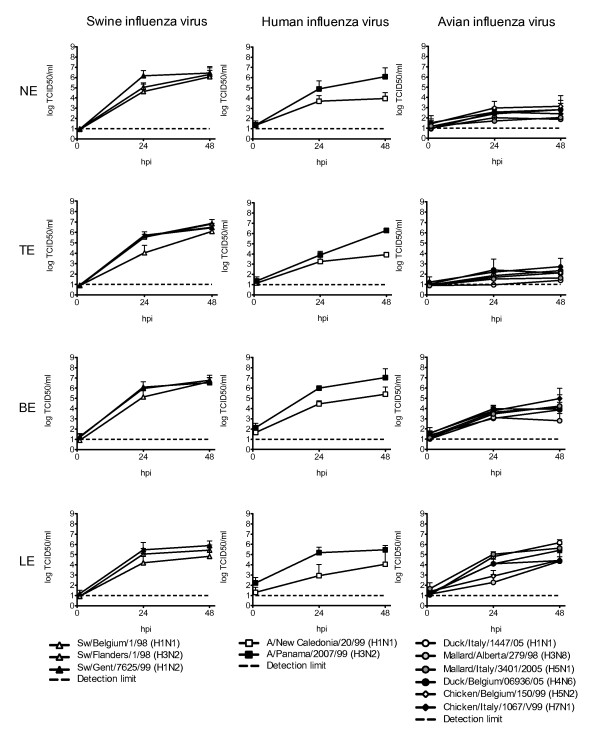
**Virus yields, expressed as log TCID_50_/ml, in the supernatant of the explants**. Virus titers were determined at 1, 24 and 48 hpi. Each row shows the results per explant system, from NE down to LE. Each column represents the host from which the different virus subtypes were isolated: pigs, humans and birds. Each value is the mean of three experiments, bars show the S.D. NE: nasal explants, TE: tracheal explants, BE: bronchial explants, LE: lung explants

All swine, human and avian isolates yielded infectious virus in the four explant systems. As shown in Figure 1, virus titers in the supernatant were significantly higher at 24 than at 1 hpi. The virus titers of Chicken/Belgium/150/99 in the supernatant of fixed explants, non permissive to infection, were at or below the detection limit by 48 hpi. This indicates that the titers of the AIVs by 48 hpi, although low in NE and TE, most likely are the result of a limited replication.

#### -Swine influenza isolates-

The three porcine influenza subtypes replicated most efficiently in the NE, TE and BE with still increasing virus yields between 24 and 48 hpi. At 48 hpi there were minimal differences in virus titers between the various subtypes. In these explants, the swine influenza viruses reached higher virus titers than any of the heterologous viruses, except for A/Panama/2007/99 (H3N2). In the LE, the replication capacity of the swine influenza viruses was more similar to that of the human and avian influenza viruses and somewhat lower than in the other explants.

#### -Human influenza isolates-

The two human isolates showed a clear distinction in their replication efficiency. In the NE, TE and BE A/Panama/2007/99 (H3N2) behaved similar to the swine influenza viruses, while the virus titers of A/New Caledonia/20/99 (H1N1) were in between those of the swine and avian strains. The virus titers of both subtypes were highest in the BE and, as for the swine influenza viruses, lower in the LE.

#### -Avian influenza viruses-

Of the heterologous viruses, the group of AIVs was least successful in replication and the only one with lower virus titers at 48 hpi than at 24 hpi in some cases. The differences in titers between the avian and swine influenza viruses were most pronounced in the NE and TE. While at 48 hpi, the maximum AIV titer reached 3.1 log TCID_50_/ml in the NE, the minimum titer of the swine influenza viruses was as high as 6.1. In the BE these differences were decreasing and they were even no longer significant in the LE. Although all AIVs preferentially bind Neu5Acα2-3Gal β-HexNAc-terminated receptors, duck and chicken viruses differ by their recognition of the inner Galβ1-3HexNAc or Galβ1-4HexNAc linkages respectively [26]. Still we did not observe a clear distinction in virus yield between the examined duck and chicken viruses.

Overall, the differences between the virus yields of swine and AIVs were statistical significant in NE, TE and BE at 24 and 48 hpi and in LE at 24 hpi only. Titers of A/New Caledonia/20/99 (H1N1) were consistently lower than those of swine influenza viruses in NE, TE and BE (except at 24 hpi in the BE). In the same explants the titers of A/Panama/2007/99 (H3N2) were invariably higher than those of AIVs.

### 3. Dose response curves

**Figure 2:**
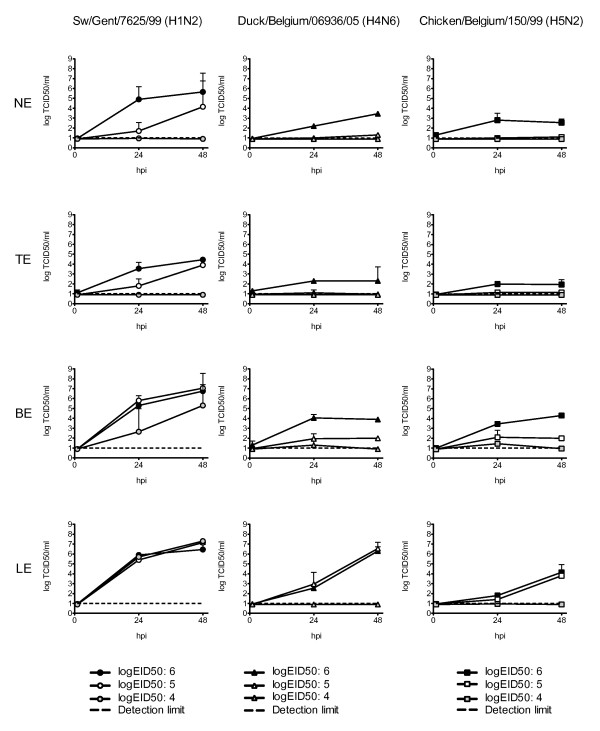
**Dose response curves for Sw/Gent/7625/99, Duck/Belgium/06936/05 and Chicken/Belgium/150/99**. Three different inoculation doses were applied: 10^6^, 10^5 ^and 10^4 ^log EID_50_. Each row represents one explant system, each column one influenza virus. The values are the mean of two experiments, bars show the S.D. NE: nasal explants, TE: tracheal explants, BE: bronchial explants, LE: lung explants

Figure 2 shows the effect on the virus yield of Swine/Gent/7625/99 (H1N2), Duck/Belgium/06936/05 (H4N6) and Chicken/Belgium/150/99 (H5N2) after inoculation with 10- and 100-fold lower doses (5 and 4 logEID_50 _respectively) than in the principal experiment. The reduction of the inoculation dose clearly had more effect on the AIVs than on the swine influenza virus. Inoculation of AIVs at 10^4 ^EID_50 _did not result in infection of the explants (titers below the detection limit), while for swine influenza viruses this was only true for NE and TE. In the BE and LE there was a limited or no reduction of the swine influenza virus yield respectively.

A 10-fold increase of the inoculation dose (10^5 ^EID_50_) of AIVs still failed to infect NE or TE. Detectable virus titers were reached in the BE and similar titers as those obtained with the highest inoculation dose in LE. The same dose of swine influenza virus resulted in infection of all explant systems by 48 hpi at levels (almost) identical to the original 10^6 ^EID_50 _dose. The slope of the virus yields between 1 and 24 hpi was remarkably less steep in NE and TE than for the highest inoculation dose.

### 4. Influenza A nucleoprotein detection

**Figure 3:**
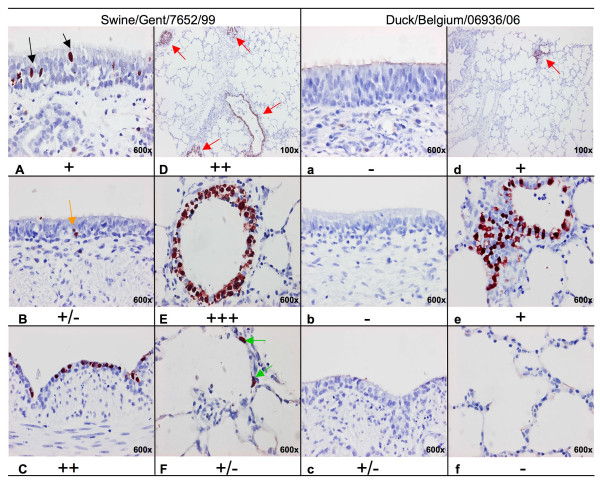
**Immunohistochemical analysis of infected cells**. Nasal (A, a), tracheal (B, b), bronchial (C, c) and lung (D→F, d→f) explants at 48 hpi inoculated with Swine/Gent/7625/99 (H1N2) (A→F) and Duck/Belgium/06936/06 (H4N6) (a→f) were analyzed. In the nasal (A: black arrow) and tracheal (B: orange arrow) explants, single swine influenza virus positive cells were diffusely spread while no avian influenza virus positive cells were present (a, b). Swine influenza virus positive cells were also found as a continuous line in bronchial epithelium (C), as multiple foci in the bronchioles (D: red arrows, E) and as single alveolar cells (F: green arrows) in lung explants. Avian influenza viral antigen-positive cells were limited to bronchiolar epithelium in lung explants (d: red arrows, e). Symbols underneath the pictures give the results for the semi-quantitative analysis of influenza virus positive cells by IF. -: no virus positive epithelial cells, +/-: single positive cells covering <10% of the epithelium, +: between 11 and 40% of the epithelium is positive, ++: between 41 and 70% of the epithelium is positive, +++: between 71 and 100% of the epithelium is positive.

An overview of the results is shown in Figure 3. Generally, cells positive by IHC displayed an intense brown intranuclear staining. They were identified in all the explant systems inoculated with the swine influenza virus (H1N2) and only in LE with the AIV (H4N6). Swine influenza virus positive cells in NE and TE were limited to diffusely spread single cells in basal and apical layers of the epithelium with distinctly more positive cells in the NE than in the TE. In the BE the level of infection was higher than in NE and TE, with up to 100% of the epithelium staining positive. Additionally the BE epithelium showed reactive atypia changing to a monolayer with few ciliated cells. Many swine influenza positive cells were also found in the LE. These contained groups of positive epithelial cells or an entirely positive epithelial lining in large and small bronchioles and, rarely, single positive alveolar cells. Detection of AIV positive cells was limited to the bronchioles of LE, with fewer foci and numbers of positive cells than for swine influenza viruses. Semi-quantitative analysis of the IF stainings confirmed these findings, as presented by the symbols in Figure 3.

### 5. Receptor expression

To determine the Sia receptor distribution in the pig from the nasal mucosa down to the alveoli we performed lectin histochemistry. Considering the results by van Riel et al. [27] on the pattern of viral attachment (PVA) of human and AIVs in pig tissues, we focused on the expression in epithelial cells and glands of NE, TE and BE and in bronchioles and alveoli of LE. An overview of the results is shown in Table 2.

**Table 2:** Summary of the lectin binding intensities of *Sambucus nigra *agglutinin (SNA) and *Maackia amurensis *agglutinin I and II (MAL-I and MAL-II) in the porcine respiratory explants

		SNA	MAL-I	MAL-II
NE	Epithelium	++	-	+/-
	Glands	+/-	+	+
TE	Epithelium	++	-	+/-
	Glands	+	+/-	-
BE	Epithelium	++	-	+/-
	Glands	++	+/-	-
LE	Bronchioles	++	-	+
	Alveolae	+	-	+

NE: nasal explants, TE: tracheal explants, BE: bronchial explants, LE: lung explants,

-: no binding, +/-: rare binding, +: moderate binding, ++: abundant binding

**Figure 4:**
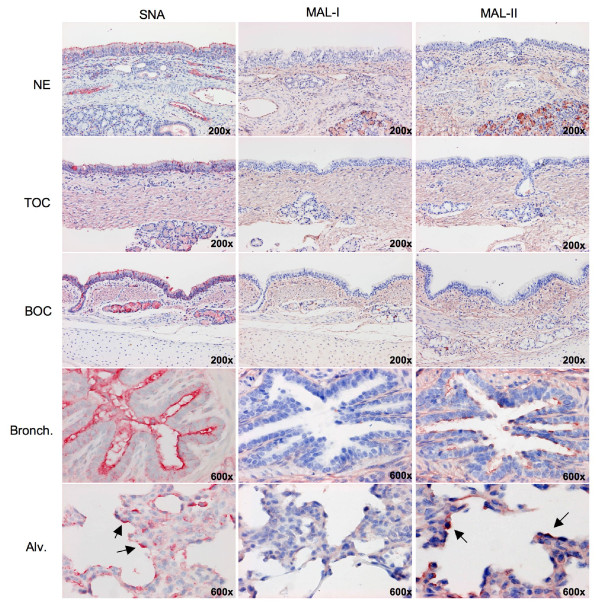
**Tissue binding of Sambucus nigra agglutinin (SNA), Maackia amurensis agglutinin I (MAL-I) and Maackia amurensis agglutinin II (MAL-II) in the different explant systems**. SNA binding (first column) was abundant in the epithelium of nasal (NE), tracheal (TE) and bronchial explants (BE) and in the epithelium of bronchioles (Bronch.), but moderate at the level of the alveolae (Alv.). MAL-I binding to epithelial cells was absent to rare in all explants systems (second column). MAL-II binding (third column) was rare in the epithelium of NE, TE and BE. At the level of the bronchioles and the alveolar tissue, it became moderate to abundant (as indicated by the black arrows).

Both α2-3- and α2-6-galactose linked Sia receptors were detected in the epithelium of the respiratory tract, but they displayed a very distinct distribution pattern. SNA binding (specific toward α2-6-galactose linked Sia) was abundant from the nasal epithelium down to the bronchioles, and more moderate in the alveoli (Figure 4). The MAL-I and MAL-II isotypes, which identify Neu5Ac(α2-3)-Gal(β1-4)-GlcNAc and Neu5Ac(α2-3)-Gal(β1-3)-GalNAc respectively [28], gave very different results. While MAL-I binding was absent in all epithelial cells, MAL-II binding was rare in nasal, tracheal and bronchial epithelium and moderate in bronchioles and alveoli. At the level of the glands, SNA binding intensity gradually increased from the NE towards the BE. On the contrary, MAL-I and MAL-II were only binding in the glands of NE at a moderate level.

**Figure 5:**
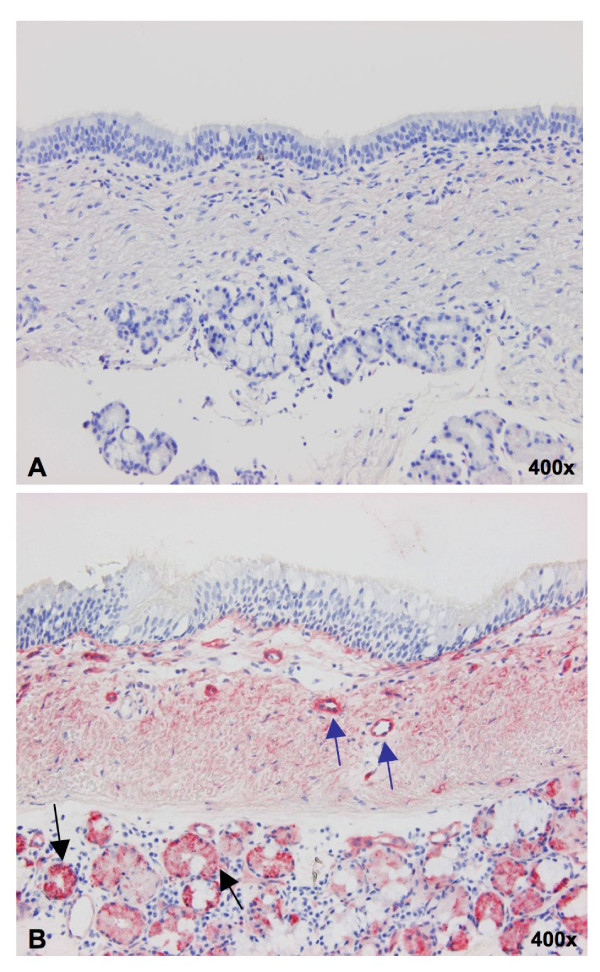
**Comparison of binding with digoxigenin-conjugated MAA in paraffin sections (A) and cryosections (B) of the porcine trachea**. Only the cryosections showed clear positivity in the glands (black arrows) and the small blood vessels (blue arrows), while paraffin sections were completely negative.

Since our findings of lack of binding with MAL-I and -II in the trachea were in disagreement with previous reports of Ito et al. [14] and Suzuki et al. [15], we tried to find an explanation for the discrepant results. Both used acetone fixed tracheal cryosections and digoxigenin labeled MAA (Dig-MAA). Duck intestines were used as a positive control. Therefore, we compared Dig-MAA binding on acetone fixed cryosections of the trachea with that on paraffin sections of paraformaldehyde fixed tissues. The frozen tissues still showed no binding of MAA to the tracheal epithelium but more positive binding to the submucous glands and to blood vessels (Figure 5).

**Figure 6:**
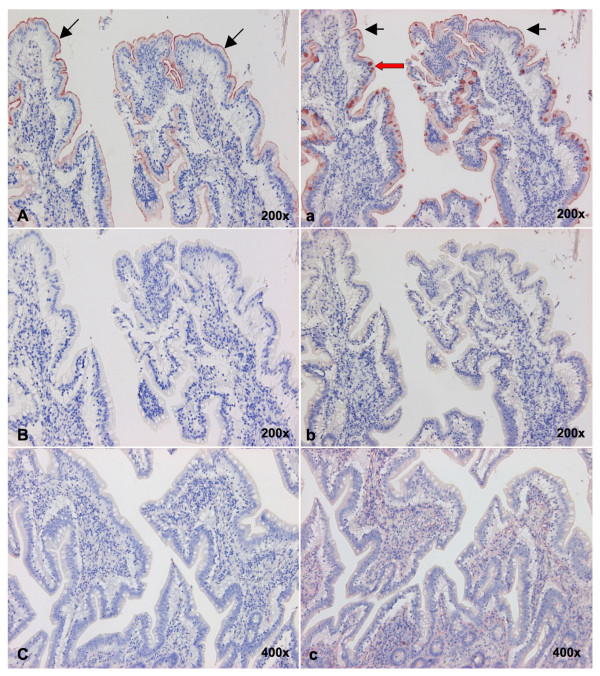
**Influence of the conjugation method of MAL-I and -II lectins on the staining intensities in duck small intestines**. Biotinylated MAL-I (A) and MAL-II (a) both resulted in epithelial cell binding (black arrows), but MAL-II (a) was additionally staining the goblet cells (red arrow). For both lectins binding was abolished by sialidase treatment of the sections (B, b). Digoxigenin labelled MAL-I (C) and MAL-II (c) failed to bind to the same tissues.

Because our MAL lectins were biotinylated instead of digoxigenin labeled we also wanted to exclude that the different conjugation method was the cause of the negative binding in the porcine trachea. For that reason we compared the binding of biotinylated MAL-I and -II with digoxigenin labeled MAL-I and -II in duck intestines. This tissue is traditionally used as a positive control because it only expresses Siaα2-3 Gal linkages. The digoxigenin labeled MAL-I and II, as shown in Figure 6 panel C and c respectively, gave no binding. The biotinylated MAL-I and -II were both binding to the intestinal epithelium but in a different pattern. The MAL-I (panel A) bound only to the apical surface of the epithelium, while MAL-II (panel a) also bound to the mucus of the goblet cells. The binding was shown to be specific, since it was abolished when the sections were pretreated with neuraminidase (panels B and b). In the porcine trachea there was no binding of either biotinylated nor digoxigenin labeled MAL.

## Discussion

We have confirmed the susceptibility of porcine respiratory tissues to infection with a range of AIVs. These AIVs replicated clearly less efficiently in tissues of the upper (nasal and tracheal) than in the lower (bronchi and alveolar) respiratory tract. This was associated with a paucity of α2,3-linked Sia receptors in the nose and trachea.

The relatively low AIV titers in porcine NE and TE may in part explain why experimental pig-to-pig transmissions of AIVs have failed so far [22,23]. This hypothesis is further strengthened by the results of our dose response experiments, in which a 10-fold reduction of the inoculation dose of AIVs completely abolished infection in NE and TE. A similar 10-fold reduction of the inoculation dose of a swine influenza viruses did not eliminate infection, indicating that the predominant distribution of an appropriate receptor is indeed an important determinant for cell tropism [29]. Wild birds infected with low pathogenic AIVs mainly excrete the virus via fecal and oculonasal discharges, while aerosol transmission is much less important [30]. We therefore speculate that a successful infection of the porcine upper respiratory tract (URT) with AIVs requires exposure to feces or fecal contaminated material with high virus concentrations. However, the likelihood that an entirely AIV successively infects several pigs, allowing a gradual adaptation to a mammalian host by point mutations, was probably overestimated in the past.

Since the infectivity pattern in our *in vitro *system is consistent with previous studies on avian, human and swine influenza virus attachment and replication, it is a valuable alternative to *in vivo *experiments. Two recent pig infection studies [31,32] clearly showed a lower replication efficiency for AIVs than for swine influenza viruses throughout the porcine respiratory tract. In both studies the AIVs replicated better in the lower (LRT) than in the upper respiratory tract (URT), but this was also the case for the swine influenza viruses. The latter finding contrasts with our *in vitro *system, in which swine influenza viruses reached lower titers in LEs than in NEs. This is most likely due to the presence of fewer virus-susceptible cells in LEs compared to a same surface area in NEs.

Our results on lectin binding intensities were not entirely in line with previous studies. We confirmed the abundant expression of α2-6-linked Sia receptors in the trachea as well as in other parts of the respiratory tract, but α2-3-linked Sia receptors were only detected in the bronchioles and alveoli, with moderate intensity. Overall we showed the Sia receptor distribution in the pig tissues to be similar to that in humans [33-35]. Even when repeating the methods of Ito et al. [14], no α2-3-linked Sia receptors could be identified in the trachea. Van Riel et al. [27] have previously studied the pattern of virus attachment in porcine respiratory tissues using labeled avian and human influenza viruses. Human viruses attached to many cells in the trachea, bronchus, bronchioles and to a moderate number in the alveolae, which is in agreement with our SNA binding intensities. As for the avian viruses, there was a lack of binding in trachea and bronchus, but increased binding in the lung, which is in accordance with our MAL-II staining. These patterns of viral attachment therefore agree with our lectin stainings, and they dispute the much cited study by Ito et al. It is of interest to note that chicken and duck influenza isolates are known to prefer SAα2,3-Gal β1,4 Glc NAc (as recognized by MAL-I) and SAα2,3-Gal β1,3 Gal NAc (as recognized by MAL-II) respectively [26,36]. As MAL-I binding in all the explant systems was negative, we would expect a reduced replication potential of the chicken isolates, which was not the case. All this fits with the hypothesis of Guo et al. [37], who state that Sia are necessary but not sufficient to act as the cellular receptor. This could also explain for the examples where influenza virus entry did not seem to be affected by a depletion of cell surface Sia [38,39].

Even within one group of heterologous viruses, some possess a higher infectivity than others. A/New Caledonia/20/99 (H1N1) had a 2 log_10 _lower viral yield than A/Panama/2007/99 (H3N2). Though both viruses are expected to have mainly a Siaα2-6 tropism [38], Wan and Perez [16] have suggested a dual receptor specificity (for both human- and avian-like receptors) for A/New Caledonia/20/99 (H1N1) and a strict Siaα2-6 preference for A/Panama/2007/99 (H3N2). To assess whether certain viruses are more likely to undergo interspecies transmissions, molecular differences responsible for this difference in infectivity will have to be identified.

## Conclusions

In this study we successfully developed an *in vitro *model that covers the entire porcine respiratory tract and is permissive to influenza virus replication in a similar way as *in vivo*. The infectivity of AIVs was shown to be low in the URT, while the pattern of human influenza viruses more closely resembled that of swine influenza viruses. These findings correlated with the Sia receptor distribution in the pig tissues, which was shown to be similar to that in humans. Consequently, the classical hypothesis on the unique role of the pig as a mixing vessel, based on the abundant expression of both α2,3-linked and α2,6-linked Sia receptors in the trachea, no longer stands. Simultaneous presence of human- and avian-type receptors has also been identified in humans [34,35,40], ducks and quail [16,41], and Thompson and colleagues [39] have generated data indicating that co-infection of human ciliated epithelial cells with human and avian influenza viruses could occur. Therefore, more detailed studies on the mechanism and on predisposing factors of reassortment are required to asses the true role of the pig.

## Methods

### 1. Animals

Five 6-week-old pigs from a high health status farm that was negative for influenza A viruses were used. The animals were housed together in a HEPA-filtered experimental unit with *ad libitum *access to water and food. At arrival they were treated intramuscularly with ceftiofur (Naxcel^®^, Pfizer-1 ml/20 kg body weight) to clear the respiratory tract from possible infections with *Actinobacillus pleuropneumoniae, Pasteurella multocida, Haemophilus parasuis *and *Streptococcus suis*. Two days later they were euthanized by intravenous administration of thiopental (Penthotal^®^, Kela-12.5 mg/kg body weight) and exsanguinated.

### 2. Isolation and culture of the respiratory explants

To cover both the upper and lower respiratory tract, four different systems were used: nasal (NE), tracheal (TE), bronchial (BE) and lung explants (LE).

#### -Nasal explants-

The NE were cultivated according to the air-liquid interface principle. NE were prepared as described by Glorieux et al. [42]. In short, the respiratory mucosa was carefully stripped from the medial side of the ventral turbinates and from the nasal septum. This tissue was cut in squares of 25 mm^2 ^each, which were transferred to fine meshed gauzes in 6-well plates with the epithelium facing up. Each well contained two ml of medium ((50% DMEM (Gibco)/50% RPMI (Gibco), penicillin 100 U/ml (Gibco), streptomycin 100 μg/ml (Gibco), gentamycin 0.1 mg/ml (Gibco), glutamine 0.3 mg/ml (BDH Biochemical)) so the epithelium was slightly immersed in fluid. Explants were cultured in an incubator at 37°C and 5% CO_2_.

#### -Tracheal organ cultures-

The trachea was excised distal from the larynx and proximal to the bifurcation. This part was divided in two by a sagittal incision and both halves were pinned onto a sterile board so the adventitia and cartilage could be removed. The remaining tissue (mucosa with some submucosa) was then cut in pieces of 25 mm^2 ^and processed similar to the nasal mucosa. Cultivation also took place following the air-liquid interface principle.

#### -Bronchial organ cultures-

The left lung was removed from the thorax and placed into transport medium (phosphate buffered saline (PBS), penicillin 1000 U/ml (Gibco), streptomycin 1 mg/ml (Gibco), gentamycin 0.5 mg/ml (Gibco), amphotericin B 5 mg/ml (fungizone^®^, Bristol-Myers)). Next the surrounding lung tissue was manually dissected out until only the bronchial tree remained. Bronchial rings of approximately two mm in diameter and three mm long were cut. These rings were transferred to 16 ml capped culture tubes containing one ml of medium (MEM (Gibco), penicillin 100 U/ml (Gibco), streptomycin 100 μg/ml (Gibco), kanamycin 1 μg/ml (Gibco), glutamine 0.3 mg/ml (BDH Biochemical), HEPES 0,02 M/100 ml (Gibco)). To imitate the in vivo situation, explants were alternately exposed to air and medium by putting them at 37°C in a slowly turning device (0.5 turn/minute) for rotating culture tubes.

#### -Lung explants-

Thin LE were obtained following a technique described earlier for rat lung explants [43], with slight modifications. We opened the thorax in the ventral midline and placed a ligature on the left trachea bifurcation. Two canules, each connected to a 20 ml syringe, were advanced down the right trachea bifurcation. One syringe contained cold PBS, the other air. As such the right lung was simultaneously perfused and aerated in situ. In the laminar flow the right apical lobe was fully expanded by inflating a 1% agarose solution. The agarose (type VII-A low gelling temperature, Sigma) had been dissolved in white PBS, autoclaved, microwave heated and cooled down to 37°C. The expanded lung was placed at 4°C for 10 minutes in a sterile container until the agarose solidified. This tissue was then cut up in cubes with a cross section of one cm^2^, which were transferred to a 20 ml syringe with seven ml of 4% agarose. After replenishing the syringe with more agarose it was placed at 4°C for 15 minutes. The embedded lung tissue was cut in slices of one mm thick, using a cryotome blade. These slices were trimmed until they had a surface of 25 mm^2 ^and incubated overnight in 24-well plates with one ml of medium (DMEM (Gibco), bovine insulin 2.5 μg/ml (Sigma), hydrocortisone 0.5 μg/ml (Sigma), vitamin A 0.5 μg/ml (Sigma), gentamycin 0.1 mg/ml (Gibco)) at 37°C and 5% CO_2_. After 24 hours the explants were thoroughly washed with warm PBS to remove the remaining agarose. Finally they were transferred to 6-well plates with two ml of medium and cultured at 37°C and 5% CO_2_.

### 3. Analysis of viability

To evaluate virus yields over time we had to confirm that fluctuations in virus titers did not result from a decreased explant viability.

Nasal and tracheal explants were checked daily for ciliary beating by light microscopy.

At 0, 24, 48, 72 and 96 hours post culture two explants of each system were collected. One was used to determine the percentage of necrotic cells by an EMA staining (Invitrogen), the other to determine the percentage of apoptotic epithelial cells using an "In Situ Cell Death Detection Kit" (Roche) [44]. The latter is based on Terminal deoxynucleotidyl transferase mediated dUTP Nick End Labelling (TUNEL) to detect DNA strand breaks. The EMA staining was performed on non fixed tissues, the TUNEL staining on cryostat sections. From each explant 12 cryosections, dispersed over the entire sample, were cut. For each section the positive cells within one ad random selected microscopic field (magnification 1000×) were counted. The number of epithelial cells in the NE, TE and BE as well as the total number of cells in the LE were determined by staining the nuclei with Hoechst (1:100 diluted in ultra pure water (U.P.)) (Invitrogen).

#### -EMA-

The explants were transferred to a 24-well plate and washed once with medium. They were incubated in the dark with 300 μl EMA (1/20 diluted in medium) for one hour at 4°C. Next they were exposed to a bright light source for 10 minutes. After three more washes with medium, the explants were embedded in methylcellulose (Methocel^®^, Fluka) and preserved at -70°C. Afterwards six μm thick sections were methanol fixed during 20 minutes at -20°C and counterstained with Hoechst (1:100 in U.P.) (Invitrogen).

#### -TUNEL-

The TUNEL reaction was performed according to the manufacturer's instructions. The explants were embedded in methylcellulose, cut into 12 slices of six μm thick, methanol fixed and counterstained with Hoechst.

### 4. Viruses, inoculation and evaluation of virus replication

Three porcine, two human and six AIVs were used (overview Table 3). The human and porcine influenza strains were representatives of viruses that are currently widespread in the human or swine population. The avian viruses were low pathogenic isolates from both *Galliformes *and *Anseriformes *and belonged to different HA subtypes. At least three repeats were conducted for each explant system and each virus subtype.

**Table 3:** Summary of the influenza viruses used for inoculation of the explants

Influenza virus	Number of passages in embryonated eggs
Swine influenza viruses:	
Sw/Belgium/1/98 (H1N1)	3
Sw/Flanders/1/98(H3N2)	3
Sw/Gent/7625/99 (H1N2)	3

Human influenza viruses:	
A/New Caledonia/20/99 (H1N1) *	4
A/Panama/2007/99 (H3N2) *	7

Low pathogenic AIVs:	
Duck/Italy/1447/05 (H1N1)^§^	4
Mallard/Alberta/279/98 (H3N8)^†^	3
Duck/Belgium/06936/05 (H4N6) ^‡^	3
Chicken/Belgium/150/99 (H5N2)^‡^	3
Chicken/Italy/1067/V99 (H7N1) ^§^	4
Mallard/Italy/3401/2005 (H5N1) ^§^	3

These viruses were kindly provided to us by:

* Alan Hay, National Institute for Medical Research, London, U.K.

† Robert Webster, St. Jude Children's Research Hospital, Memphis, Tennessee, U.S.A.

‡ Thierry van den Berg, Veterinary and Agrochemical Research Centre, Brussels, Belgium

§ Ilaria Capua, Instituto Zooprofillatico delle Venezie, Legnaro, Padova, Italy

After 24 hours of culture and one washing step with PBS, the explants were inoculated with 10^6 ^EID_50 _virus in a volume of 600 μl. For this purpose, NE, TE and LE were transferred to 24-well plates while the BE remained in the cell culture tubes. One hour of incubation with the inoculum at 37°C was followed by three subsequent washing steps with 0.5 ml warm PBS so non-attached viruses were removed. Next the explants were placed back in the original 6-well plates or culture tubes with 2.3 ml (NE, TE and LE) or 1.3 ml (BE) of new medium.

To assess virus yields, 300 μl of supernatant was collected at 1, 24 and 48 hours post inoculation. Ten-fold serial dilutions of the supernatant were inoculated onto MDCK cells grown in 96-well plates. These plates were incubated for seven days at 37°C with 5% CO_2 _and checked for CPE. The presence of virus replication in each well was confirmed by an immunocytochemical staining, which was analyzed by light microscopy. The cells, fixed with paraformaldehyde 4% for 10 minutes at room temperature, were incubated with mouse anti-NP monoclonal HB-65 antibody (1:50, ATCC) for two hours. Subsequently, incubation with horseradish peroxidase-conjugated goat anti-mouse polyclonal antibody (1:200, Dako) for one hour was followed by a development step with H_2_0_2 _as substrate and 3-amino-9-ethyl-carbazole (AEC) as precipitating agent. Virus titers were calculated by the method of Reed and Muench and expressed as TCID_50_/ml. Statistical analysis to compare the titers of the avian, the swine and human viruses in Figure 1 was carried out using the Kruskal-Wallis test with a 95% confidence interval (p < 0.05). The avian and swine viruses were compared as groups at 24 and 48 hpi, the human viruses were compared separately because of the consistent differences in virus yield between the 2 subtypes.

Because the virus titers obtained with AIVs were frequently low, additional controls were performed to confirm that these titers resulted from productive virus infection rather than from the release of input virus immediately after the attachment step. To this purpose a parallel experiment was performed with explants that only allow attachment of the virus and no virus entry or subsequent steps [45]. One NE, TE, BE and LE were prepared as described above. After 24 hours they were fixed in one ml of 1% paraformaldehyde at 4°C for one hour. Next they were extensively washed with PBS and inoculated with 600 μl 10^6 ^EID_50 _Chicken/Belgium/150/99 (H5N2). After one hour of incubation at 37°C the inoculum was removed, the explants were washed three times with PBS and the medium was renewed. Supernatant was again collected at 1, 24 and 48 hpi.

### 5. Evaluation of the dose response

Additionally we wished to examine the effect of the inoculation dose on the yield of swine and AIVs at different levels of the respiratory tract. Therefore NE, TE, BE and LE were inoculated with Swine/Gent/7625/99 (H1N2), Duck/Belgium/06936/05 (H4N6) and Chicken/Belgium/150/99 (H5N2) at three different doses: 10^6, ^10^5 ^and 10^4 ^EID_50 _virus in a volume of 600 μl. Collection and titration of the supernatant was performed as described above. Each condition was repeated twice.

### 6. Influenza A nucleoprotein detection

Since virus titrations of the supernatant do not provide information on the number or type of infected cells, we fixed the explants inoculated with Swine/Gent/7625/99 (H1N2) and Duck/Belgium/06936/05 (H4N6) at 48 hpi to perform immunohistochemistry (IHC) and immunofluorescense (IF).

IHC was carried out on formalin fixed (for 24 hours) and paraffin embedded explants. Ten consecutive sections of 4 μm thick were cut in six different areas of each explant. Because formalin-fixation can cause protein cross-linking, antigen retrieval (AR) was applied to stain the viral nucleoprotein. Enzyme-induced AR was accomplished by incubating the deparaffinized and rehydrated sections with 0,1% pronase (Roche) at 37°C for four minutes. Endogenous peroxidase and biotin activity were blocked by incubation with 3% H_2_O_2 _and the biotin/avidin blocking kit (Vector) at room temperature respectively. Incubation with the anti-influenza A nucleoprotein monoclonal antibody at a 1:400 dilution was performed at room temperature for 90 minutes. Slides were rinsed three times in 0.05 mol/L Tris-buffer and incubated with biotin-conjugated rabbit anti-mouse immunoglobulin at a 1:100 dilution (Dako) for 30 minutes at room temperature. After another wash they were incubated with the ABC-complex for 30 minutes, developed with AEC (Vector developing kit) and counterstained with hematoxylin.

To exclude that AR gives rise to false positive results, we compared the localization of virus positive cells after IHC with those after IF. Therefore similar explants were embedded in Methocel^® ^(Sigma), frozen at -70°C and entirely cut in sections of 8 μm thick. These sections were fixed in methanol for 10 minutes at -20°C. Since this fixation method only causes precipitation of proteins, AR was not required. The anti-influenza A nucleoprotein monoclonal antibody was incubated at a 1:50 dilution followed by FITC labeled goat-anti mouse IgG (Molecular Probes) at a 1:200 dilution, both for one hour at 37°C.

Semi quantitative information on the infectivity was obtained by IF on cryosections, where the following scoring system was applied: -: no virus positive epithelial cells, +/-: single positive cells covering <10% of the epithelium, +: between 11 and 40% of the epithelium is positive, ++: between 41 and 70% of the epithelium is positive, +++: between 71 and 100% of the epithelium is positive.

### 7. Lectin histochemistry

The distribution of α2-3- and α2-6-galactose linked Sia receptors in explants, 24 hours post culture, was detected by lectin histochemistry. The tissues had been fixed in 4% buffered formalin during 24 hours and were paraffin embedded. In an identical manner fresh nasal, tracheal, bronchial and lung tissues were analyzed to check for the effects of cultivation on receptor expression. Because formalin fixation can cause protein cross-linking, thereby hiding antigenic sites, antigen retrieval (AR) was applied. Nicholls et al. [37] have previously shown that heat induced AR by microwaving the samples in 10 mM citrate buffer pH 6.0 at 95°C for 15 minutes is the optimal method for the retrieval of Sia receptors. To exclude false positive results by AR, some control sections were pretreated with neuraminidase removing the Sia. In these stainings we obtained negative results, showing that the AR unmasked only the epitope of interest.

Duck intestines, which only contain Siaα2-3Gal linkages, were used as a control for the specificity of the MAA and SNA lectins.

#### -Expression of α2-6 linked Sia-

The α2-6 distribution was examined using a digoxigenin labelled *Sambucus nigra *agglutinin (SNA) used at a 1:200 dilution (Roche). Sections were incubated for one hour at room temperature. Subsequently, the slides were incubated with 1:200 sheep alkaline phosphatase conjugated anti-digoxigenin Fab fragments (Roche) and developed with New Fuchsin (Dako).

#### -Expression of α2-3 linked Sia-

Two different isoforms of the *Maackia amurensis *agglutinin (MAA), MAL-I and MAL-II that both bind to the α2-3-linked Sia moiety of the receptor, were used. They do differentiate though at the next glycoside level between galactose β1-4- or β1-3-linkages respectively [46]. The biotinylated MAL-I and -II (Vector Laboratories), both 1:200 diluted, were incubated overnight at 4°C. Staining was developed with a strep-ABC complex 1:100 (DakoCytomation) and an AEC-substrate kit (Vector Laboratories). Subsequent sections were also stained with digoxigenin conjugated MAA from Roche, as used by Ito and colleagues (16), together with digoxigenin conjugated MAL-I and MAL-II. The latter were obtained using the Dig Conjugation Kit (Roche) and aimed to examine if the conjugation method can influence the lectin binding.

## Competing interests

The authors declare that they have no competing interests.

## Authors' contributions

SGMVP carried out the explant work and the IF, performed the statistical analysis and drafted the manuscript. JMN participated in immunohistochemistry and lectin histochemistry, as well as in the interpretation of the results. HJN helped to establish the explant system and to interpret the data. KVR participated in conceiving the study and completed the manuscript. All authors read and approved the final manuscript.
